# Targeted Modulation of the Gut Microbiota in Obesity: From Dietary Interventions to Bacteriophage-Based Precision Therapies

**DOI:** 10.3390/nu18213508

**Published:** 2026-10-25

**Authors:** Sthefhany Nohemí Rodríguez-Arellano, Jean Pierre González-Gómez, José Roberto Aguirre-Sánchez, Bruno Gomez-Gil, Elisa Barrón-Cabrera, Cristobal Chaidez, Marcela de Jesús Vergara-Jiménez

**Affiliations:** 1 Facultad de Ciencias de la Nutrición y Gastronomía, Universidad Autónoma de Sinaloa, Culiacan 80019, Sinaloa, Mexico; sthefhanyrodriguez.fcng@uas.edu.mx (S.N.R.-A.); elisabarron@uas.edu.mx (E.B.-C.); 2 Laboratorio Nacional para la Investigación en Inocuidad Alimentaria (LANIIA), Centro de Investigación en Alimentación y Desarrollo, A.C. (CIAD), Culiacan 80110, Sinaloa, Mexico; jean.gonzalez@ciad.mx (J.P.G.-G.); jose.aguirre@ciad.mx (J.R.A.-S.); 3 Centro de Inteligencia Artificial Aplicada e Innovación Tecnológica, Universidad Autónoma de Sinaloa, Culiacan 80019, Sinaloa, Mexico; 4 Centro de Investigación en Alimentación y Desarrollo, A.C. (CIAD), Unidad Mazatlán en Acuicultura y Manejo Ambiental, Mazatlan 82112, Sinaloa, Mexico; bruno@ciad.mx

**Keywords:** obesity, gut microbiota, gut dysbiosis, inflammation, precision microbiome modulation, phage therapy

## Abstract

Obesity is a complex chronic disease associated with metabolic and inflammatory alterations in which the gut microbiota has emerged as an important contributing factor. Obesity-associated gut dysbiosis involves not only changes in microbial composition but also alterations in microbial functions and metabolites that may affect intestinal barrier integrity, immune regulation, adipose tissue metabolism, insulin sensitivity, and glucose homeostasis. Current microbiota-modulating strategies, including dietary interventions, prebiotics, probiotics, synbiotics, and fecal microbiota transplantation, can influence the gut microbial ecosystem; however, their effects are often highly variable among individuals and generally lack the specificity required to selectively target disease-associated bacterial populations. These limitations have increased interest in precision microbiota modulation, which aims to selectively modify microbial populations, functions, or products while minimizing unnecessary alterations to the remaining microbial community. In this context, bacteriophages represent a promising biological tool because of their ability to specifically target bacterial hosts, offering the possibility of selectively reducing bacterial populations associated with obesity-related metabolic and inflammatory disturbances. Experimental studies and early human trials have shown that bacteriophage-based interventions can modulate gut microbial communities and influence inflammatory responses, supporting their potential relevance to obesity-associated chronic low-grade inflammation. However, no published clinical trials specifically evaluating targeted bacteriophage therapy for obesity treatment were identified, and its efficacy for obesity management remains unestablished. Further research is needed to identify and validate bacterial targets, optimize phage selection and cocktail design, and evaluate phage stability, bacterial resistance, immunomodulatory effects, safety, and host–microbiota interactions before clinical translation can be considered.

## 1. Introduction

Obesity is a major global chronic health condition involving excessive adipose tissue accumulation that can compromise health. Its etiology is multifactorial, reflecting interactions among genetic, environmental, and lifestyle-related factors, together with changes in the gut microbiota [1]. Among environmental factors, lifestyle encompasses dietary practices, physical activity, stress management, sleep patterns, and social relationships. Over recent decades, the growing consumption of ultra-processed foods, together with increasingly sedentary behaviors, has contributed to the worldwide increase in overweight and obesity [2,3]. The interaction among these factors not only contributes to the development of obesity but also promotes metabolic and immune alterations that complicate its prevention and treatment [4].

In addition to its complex etiology, obesity is associated with multiple complications that substantially increase its impact on health. Excess adiposity increases the risk of developing chronic non-communicable diseases [5], particularly cardiovascular diseases, such as coronary heart disease and stroke, as well as diabetes, kidney, liver, and respiratory diseases, musculoskeletal and neuropsychiatric disorders, and several types of cancer. These comorbidities contribute substantially to the increased morbidity and mortality associated with obesity [6,7].

The magnitude of this problem continues to increase. Currently, more than one-third of the global population is affected by overweight or obesity, and if current trends persist, this figure is projected to exceed 4 billion people by 2035, representing more than half of the global population [8,9]. Beyond its impact on health, obesity imposes a substantial economic burden, with an estimated global cost of USD 1.88 trillion. Without the implementation of effective prevention and treatment strategies, these costs are projected to increase rapidly over the coming decades, particularly in low- and middle-income countries [10,11].

Obesity management is primarily based on lifestyle modification, through interventions aimed at improving dietary habits, increasing physical activity, and incorporating cognitive–behavioral strategies when necessary [12]. When these measures fail to achieve or maintain clinically significant weight loss, they may be complemented by pharmacological treatment and, in patients with severe obesity or associated comorbidities, by bariatric surgery, which offers the greatest potential for sustained weight reduction and improvement in cardiometabolic risk among currently established interventions [13,14]. Nevertheless, despite advances in obesity treatment, therapeutic responses remain highly variable among individuals, and long-term weight loss maintenance continues to represent a major challenge [15]. These limitations reflect the multifactorial nature of obesity and indicate that current strategies do not comprehensively address all the biological mechanisms involved in its development and progression [16]. Consequently, growing interest has emerged in recent years in identifying novel therapeutic targets that may complement conventional strategies, among which the gut microbiota has gained particular relevance [17].

The gut microbiota constitutes a complex and dynamic ecosystem that participates in multiple physiological and metabolic functions of the host. Its composition is influenced by several factors, among which lifestyle, particularly diet, plays a major role [18,19]. In addition to contributing to nutrient digestion, absorption, and metabolism, the gut microbiota participates in the regulation of appetite and eating behavior through bidirectional communication along the microbiota–gut–brain axis [20,21]. Therefore, alterations in its composition or function can disrupt host immune and metabolic homeostasis, contributing to the development and progression of diseases such as obesity [22].

Recognition of the role of the gut microbiota in the pathophysiology of obesity has driven the development of various strategies aimed at modulating its composition and activity. These include probiotics, prebiotics, and fecal microbiota transplantation, which primarily seek to restore the balance of the intestinal ecosystem by enriching microorganisms considered beneficial [23,24]. However, this approach has placed less emphasis on the selective elimination of pathobionts associated with inflammatory processes and obesity-related metabolic alterations [25]. Consequently, there is a growing need for precision modulation strategies that specifically target disease-causing microorganisms while preserving the stability of the remaining microbial community [26].

In this context, the host specificity of bacteriophages offers an opportunity to modulate selected gut bacterial populations while potentially preserving much of the surrounding microbial community [27]. Accordingly, this review examines current evidence on the role of the gut microbiota in obesity, available dietary strategies for its modulation, and the potential of bacteriophage therapy as a precision tool for the treatment of this disease.

## 2. Materials and Methods

### Literature Search and Review Methodology

This manuscript was developed as a narrative and critical review of the current evidence on gut microbiota modulation in obesity, with particular emphasis on emerging bacteriophage-based precision strategies. The literature search was conducted using the scientific databases PubMed, Scopus, Web of Science, and Google Scholar. Searches were guided by the conceptual framework and objectives of the review and covered the following major topics: (i) the composition and function of the gut microbiota in obesity; (ii) mechanisms linking microbial alterations with metabolic dysfunction and inflammation; (iii) conventional microbiota-modulation strategies; (iv) precision microbiome modulation; (v) bacteriophage biology and phage–microbiota–host interactions; (vi) experimental evidence for phage-mediated microbiota modulation; (vii) challenges and limitations associated with phage-based approaches; and (viii) phage cocktail design, combination therapies, and clinical translation. Relevant publications were selected based on their relevance to the specific topics addressed, scientific contribution, and mechanistic, experimental, or translational significance. Primary research articles were prioritized when discussing experimental evidence, while relevant review and conceptual articles were incorporated to provide broader context. Reference lists of selected publications were also examined to identify additional relevant studies.

## 3. Gut Microbiota and Obesity: Current Understanding

### 3.1. Microbial Composition Shifts

The application of next-generation sequencing and bioinformatic analyses have improved the resolution with which gut microbial communities can be characterized, facilitating investigations of microbial differences associated with obesity [28]. Given the substantial interindividual variability in gut microbiota composition, studies involving larger populations are increasingly important for distinguishing reproducible obesity-associated microbial patterns from individual variation and for examining microbiota–host interactions [29].

Gut microbiota composition is shaped by the interaction of multiple factors that act from early life and continue to influence it throughout the lifespan. These include mode of delivery, dietary patterns, medication use, including antibiotics, as well as other lifestyle- and environment-related factors that can influence microbial diversity and composition [30,31]. In particular, a high-fat diet has been associated with reduced microbial diversity and alterations in bacterial populations involved in maintaining intestinal barrier integrity [32]. Likewise, physical activity may promote changes in the gut microbiota that are potentially associated with improved health outcomes [33]. Geographic factors also contribute to this variability, as distinct microbial signatures have been identified across different populations and may persist from childhood into adulthood [34]. Collectively, the interplay among these factors highlights the dynamic nature of the gut microbiota and may explain, at least in part, the heterogeneity of microbial profiles observed among individuals with obesity.

In obesity, variations in the relative abundance of the major bacterial groups that comprise the gut microbiota have been reported. Among the most represented phyla are Bacillota and Bacteroidota, which together account for approximately 90% of the gut bacterial community, followed in lower proportions by Actinomycetota, Pseudomonadota, Fusobacteriota, and Verrucomicrobiota [31,35]. These groups include genera of relevance to the gut microbiota, such as *Clostridium*, *Lactobacillus*, and *Ruminococcus* within Bacillota; *Bacteroides* and *Prevotella* within Bacteroidota; and *Bifidobacterium* within Actinomycetota [36]. The relative abundance of these bacterial groups can vary considerably among individuals with obesity, making it difficult to establish a characteristic microbial composition associated with this condition [37].

One of the earliest findings linking the gut microbiota to obesity was an altered Bacillota/Bacteroidota (B/B) ratio, historically referred to as the Firmicutes/Bacteroidetes (F/B) ratio. Pioneering studies based on 16S rRNA gene sequencing reported a higher proportion of Bacillota and a lower abundance of Bacteroidota in association with obesity [38]. These findings gave rise to the hypothesis that specific gut microbiota configurations could enhance energy extraction from dietary components that are otherwise indigestible by the host, potentially contributing to increased body fat storage [39]. Based on these observations, the B/B ratio was extensively investigated as a potential microbial signature associated with obesity; however, subsequent evidence has questioned its utility due to the lack of reproducibility across studies [40]. Furthermore, the biological interpretability of the B/B ratio is limited by its phylum-level aggregation of taxonomically and functionally diverse bacteria, which may obscure species- and strain-specific contributions to host metabolism and inflammation [41]. Systematic reviews and meta-analyses have revealed considerable heterogeneity in the findings, with studies reporting increased, decreased, or no significant differences in this ratio in relation to obesity [42,43]. These discrepancies may be related to the high interindividual variability of the gut microbiota and to factors such as diet and the characteristics of the populations studied [44], as well as differences in sequencing approaches, bioinformatic analyses, and study design [45]. Collectively, these factors hinder the identification of a universal microbial signature associated with obesity. Recent evidence regarding the association between the B/B ratio and obesity, together with relevant factors and methodological considerations for interpreting these findings, is summarized in Table 1.

Beyond global variations in gut microbiota composition, several studies have identified specific bacterial taxa associated with obesity, metabolic dysfunction, and the inflammatory processes accompanying this condition. Some bacteria, such as *Akkermansia muciniphila* [53,54,55], *Christensenella minuta* [56,57], *Parabacteroides distasonis* [58], *Blautia wexlerae* [59], *Bacteroides vulgatus* [60], and *Faecalibacterium prausnitzii* [61], have been associated with beneficial effects on different metabolic parameters, whereas *Roseburia intestinalis* [62] has shown anti-inflammatory properties linked to the regulation of immune responses. In contrast, *Ruminococcus gnavus* [63] and *Enterobacter cloacae* B29 [64] have been associated with pro-inflammatory responses and metabolic alterations. Collectively, these findings indicate that the relationship between the gut microbiota and obesity depends not only on global changes in microbial composition but also on the presence and activity of specific taxa, whose effects may vary even at the strain level (Table 2).

### 3.2. Functional Dysbiosis

In addition to differences in the abundance of specific taxa, obesity has also been associated with alterations in the functional potential of the gut microbiota [65]. A meta-analysis integrating 3329 samples from 17 countries identified 37 metabolic pathways associated with obesity and revealed differences in the functional repertoire of the gut microbiota between individuals with and without obesity. In particular, pathways related to carbohydrate metabolism and purine and pyrimidine biosynthesis were more enriched in individuals without obesity, whereas pathways related to amino acid metabolism and the biosynthesis of enzyme cofactors and peptidoglycan predominated in individuals with obesity. Moreover, although some of these alterations were reproducible across populations, others varied among the datasets analyzed, indicating that obesity-associated heterogeneity also extends to the functional potential of the gut microbiota [66].

In obesity, functional dysbiosis has also been associated with alterations in short-chain fatty acid (SCFA) profiles, particularly acetate, propionate, and butyrate [67,68]. Although these metabolites are generally associated with beneficial effects on metabolic homeostasis, some studies have reported elevated fecal SCFA concentrations in individuals with obesity, whereas others have not observed the same pattern, highlighting the complex relationship between these metabolites and the disease [69]. Furthermore, fecal SCFA concentrations do not necessarily reflect their intestinal production, as they may be influenced by factors such as microbial production, intestinal absorption, and gastrointestinal transit [70]. These differences should be considered when interpreting SCFA profiles as a functional feature of the gut microbiota associated with obesity.

Likewise, obesity has been associated with alterations in microbial bile acid metabolism [71]. The gut microbiota actively participates in bile acid transformation through processes such as deconjugation and the conversion of primary into secondary bile acids [72,73]. Because these transformations depend on different microbial enzymes, changes in gut microbiota composition and activity can modify bile acid profiles [74]. In individuals with obesity, these alterations may result in bile acid profiles that differ from those observed in individuals without obesity [75], suggesting changes in the microbial activity involved in bile acid metabolism.

Collectively, these findings indicate that obesity-associated gut dysbiosis cannot be defined exclusively by changes in the abundance of specific microorganisms [76]. The identification of alterations in different microbial functions and metabolic pathways supports the importance of considering the functional capacity of the gut microbiota in addition to its taxonomic composition [77]. Therefore, integrating both features provides a more comprehensive view of obesity-associated gut dysbiosis and contributes to a better understanding of its potential consequences for host physiology.

## 4. Mechanistic Links Between Microbiota and Obesity

The gut microbiota contributes to obesity through multiple interconnected mechanisms by which microbial communities influence host metabolic and immune homeostasis [78,79]. These mechanisms include alterations in energy metabolism, intestinal barrier integrity, immune signaling, and the production of bioactive microbial metabolites [80,81]. Changes in intestinal permeability, translocation of bacterial components, immune activation, and adipose tissue inflammation further contribute to the interaction between the gut microbiota and host metabolic tissues [82]. Collectively, these mechanisms can promote chronic low-grade inflammation and metabolic dysfunction, both of which are central to the pathophysiology of obesity [13].

### 4.1. Inflammation and Metabolic Endotoxemia

Metabolic endotoxemia is a widely investigated pathway through which the gut microbiota may contribute to obesity. It involves a persistent, moderate elevation of circulating bacterial lipopolysaccharides (LPS). High-fat diets have been associated with increased plasma LPS concentrations and the development of metabolic and inflammatory alterations characteristic of obesity [82,83].

In obesity-associated dysbiosis, impaired intestinal barrier function, together with changes in Gram-negative bacterial populations, may facilitate the passage of LPS from the intestinal lumen into systemic circulation. Once translocated, LPS interacts with LPS-binding protein (LBP), facilitating its detection through CD14 and Toll-like receptor 4 (TLR4) in immune cells and metabolically relevant tissues [84,85]. Downstream activation of nuclear factor kappa B (NF-κB) and mitogen-activated protein kinase (MAPK) signaling promotes the expression of inflammatory mediators, including TNF-α, IL-1β, IL-6, and monocyte chemoattractant protein-1 (MCP-1). The resulting inflammatory response can favor monocyte recruitment into adipose tissue and interfere with insulin signaling, thereby contributing to metabolic dysfunction [84].

Although recent studies have highlighted limitations in quantifying circulating LPS, evidence supports a broader contribution of microbiota-derived products to inflammatory signaling in obesity. In addition to LPS, other microbial structures, including peptidoglycans, flagellins, bacterial DNA, and extracellular vesicles, are recognized as potential contributors to immunometabolic activation [86].

### 4.2. Gut Barrier Integrity

The intestinal barrier regulates interactions between luminal microorganisms and host tissues [87]. Its protective function depends on the coordinated activity of the mucus layer, intestinal epithelial cells, intercellular tight junctions, antimicrobial peptides, secretory immunoglobulin A (IgA) [88], and resident immune populations [89]. Together, these components support nutrient uptake while limiting the entry of microorganisms and potentially harmful microbial products into host tissues.

However, obesity-associated dysbiosis is linked to structural and functional alterations that increase intestinal permeability, a phenomenon known as “leaky gut.” Changes in microbial community composition may contribute to this process. The depletion of beneficial butyrate-producing bacteria [90], such as *Faecalibacterium prausnitzii*, *Roseburia* spp., and *Eubacterium* spp., reduces energy supply to colonocytes and compromises the expression of tight junction proteins, including claudins, occludins, and zonula occludens-1 (ZO-1) [91]. Concurrently, reduced abundance of *Akkermansia muciniphila*, a bacterium specialized in interacting with the intestinal mucus layer, has been associated with diminished thickness and functionality of this protective barrier. Furthermore, Western diets rich in saturated fats and low in dietary fiber promote the expansion of microbial communities capable of degrading mucins [92], leading to thinning of the mucus layer and increased exposure of the epithelium to microbial components. As a result, translocation of bacterial molecules into the systemic circulation increases, providing a mechanistic link between barrier dysfunction, metabolic endotoxemia, and chronic inflammation [85].

**Figure 1:**
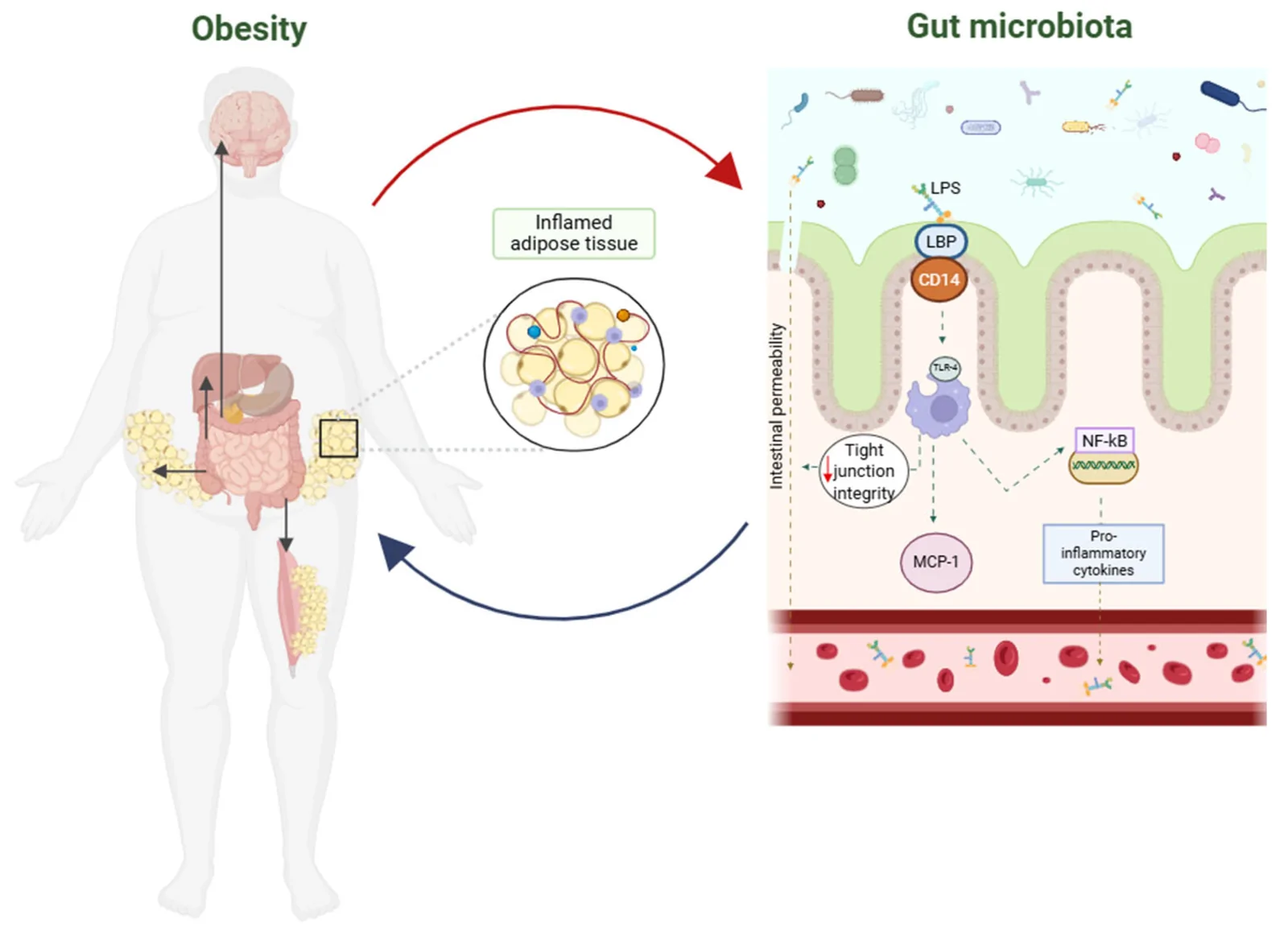
Gut microbiota–obesity crosstalk and mechanisms contributing to metabolic inflammation. The red and blue arrows illustrate the bidirectional relationship between obesity and the gut microbiota, while the arrows within the intestinal diagram represent processes associated with intestinal barrier dysfunction and inflammatory signaling. LPS: lipopolysaccharide; LBP: lipopolysaccharide-binding protein; MCP-1: monocyte chemoattractant protein-1; NF-κB: nuclear factor kappa B; TLR4: Toll-like receptor 4. Created with BioRender (https://www.biorender.com; accessed on 14 August 2026).

Loss of intestinal barrier integrity is not only a consequence of obesity but also a potential contributor to its progression. Emerging evidence further suggests that the intestinal barrier functions as a dynamic sensor integrating dietary signals, microbial metabolite production, and immune responses [93]. Thus, barrier dysfunction may represent an important interface through which alterations in the gut microbiota influence systemic metabolic homeostasis. Overall, the interplay between obesity-associated gut dysbiosis, intestinal barrier dysfunction, metabolic endotoxemia, and adipose tissue inflammation is summarized in Figure 1.

### 4.3. Microbial Metabolites and Signaling Pathways

In addition to their structural and immunological effects, gut microorganisms profoundly influence host metabolism by producing bioactive metabolites that serve as signaling molecules between microbial communities and the host.

#### 4.3.1. Short Chain Fatty Acids

Fermentation of dietary fiber and other carbohydrates that escape digestion produces short-chain fatty acids (SCFAs), with acetate, propionate, and butyrate constituting the principal products. These metabolites participate in communication between the intestinal microbiota and host tissues. Their signaling actions involve several G protein-coupled receptors, notably GPR41, GPR43, and GPR109A, through which they influence energy regulation, enteroendocrine activity, glucose handling, fatty acid oxidation, and immune function [94]. Butyrate supplies a major proportion of the energy required by colonocytes and contributes to the preservation of intestinal barrier function [91]. Propionate is involved in hepatic glucose production and appetite-related signaling, whereas acetate participates in pathways governing lipid utilization and energy balance. SCFAs can also promote the release of glucagon-like peptide-1 (GLP-1) and peptide YY (PYY), linking microbial fermentation to appetite regulation and glycemic responses [95].

Nevertheless, the relationship between SCFAs and obesity remains unclear and complex. While numerous studies have reported beneficial metabolic effects, excessive SCFA production may also enhance energy extraction from otherwise indigestible dietary components. Current evidence suggests that this relationship remains complex. Therefore, the metabolic effects of SCFAs appear to depend on the physiological context, metabolite concentrations, and the overall composition of the microbial ecosystem.

#### 4.3.2. Bile Acids

The gut microbiota plays a fundamental role in bile acid transformation [96]. Intestinal microorganisms modify bile acids through reactions such as deconjugation and conversion, generating secondary bile acids with signaling activity. Thus, bile acids participate not only in dietary lipid digestion and absorption but also in endocrine pathways involved in glucose, lipid, and energy regulation. Two principal mediators of these effects are the nuclear farnesoid X receptor (FXR) and the membrane-associated G protein-coupled bile acid receptor TGR5 [96,97]. Through FXR, bile acid signaling influences their own synthesis as well as hepatic lipid and glucose metabolism. TGR5-mediated responses include regulation of thermogenesis, energy expenditure, and glucagon-like peptide-1 (GLP-1) secretion [98].

In addition, the gut microbiota participates in shaping the composition and signaling properties of the circulating bile acid pool. Consequently, obesity-associated dysbiosis can alter the relative abundance of primary and secondary bile acids, thereby dysregulating FXR and TGR5 signaling [96,97]. These alterations have been linked to impaired glucose metabolism, increased adiposity, low-grade inflammation, and metabolic dysfunction.

Microbiota–bile acid interactions have also attracted considerable attention in the context of bariatric surgery. Experimental and clinical studies indicate that profound changes in both gut microbial communities and bile acid profiles accompany metabolic improvements following Roux-en-Y gastric bypass and sleeve gastrectomy. Increased circulating concentrations of specific secondary bile acids, together with enhanced FXR and TGR5 signaling, are thought to contribute to improvements in insulin sensitivity, energy expenditure, and glucose control observed after surgery [99,100]. These findings highlight bile acids as critical mediators of microbiota–host communication and potential therapeutic targets for obesity management.

#### 4.3.3. Indoles and Tryptophan-Derived Metabolites

Microbial metabolism of dietary tryptophan is an important mechanism that modulates host physiology through the gut microbiota. Diverse intestinal microorganisms, including members of the genera *Lactobacillus*, *Clostridium*, *Peptostreptococcus*, and *Bacteroides*, convert tryptophan into a variety of bioactive metabolites, such as indole, indole-3-acetic acid, indole-3-propionic acid, indole-3-aldehyde, and other indole derivatives [101,102].

These compounds function as signaling molecules that mediate communication between the microbiota and the host. Their biological effects are primarily mediated through activation of the aryl hydrocarbon receptor (AhR) and the pregnane X receptor (PXR), both of which play crucial roles in maintaining intestinal homeostasis and immune regulation [103]. Activation of AhR signaling promotes the production of interleukin-22 (IL-22), a cytokine involved in epithelial regeneration, antimicrobial peptide production, mucus layer maintenance, and enhancement of barrier integrity. In parallel, PXR activation contributes to epithelial protection by limiting inflammatory signaling and preserving tight junction function. Accumulating evidence suggests that alterations in microbial tryptophan metabolism contribute to obesity-associated metabolic dysfunction. Both experimental and clinical studies have reported reduced concentrations of specific indole derivatives in individuals with obesity, insulin resistance, and metabolic syndrome [104]. Decreased production of these metabolites may impair barrier function, favor microbial translocation, and exacerbate chronic low-grade inflammation, thereby contributing to the progression of metabolic disease [101].

Indole, in addition to influencing host physiology, also participates in metabolic pathways by modulating immune responses, glucose homeostasis, and adipose tissue inflammation. These effects highlight microbial tryptophan metabolism as an additional pathway linking the gut microbiota with intestinal and metabolic homeostasis. However, further studies are needed to clarify the specific contributions of individual tryptophan-derived metabolites to obesity-associated metabolic and inflammatory alterations and to determine their potential relevance as therapeutic targets [105].

### 4.4. Microbiota–Immune System Crosstalk

Interactions between intestinal microorganisms and host immunity operate in both directions and contribute to metabolic and immunological homeostasis. Microbial-derived signals participate in the development of innate and adaptive immune responses, whereas host immune mechanisms help regulate the composition of resident microbial communities and sustain their coexistence with the host. In a balanced intestinal environment, commensal bacteria support immune tolerance partly through the induction of regulatory T cells (Tregs) [106,107], the promotion of anti-inflammatory mediators such as interleukin-10 (IL-10), and the maintenance of epithelial barrier function [108].

Obesity-associated dysbiosis disrupts this homeostatic relationship, leading to persistent activation of innate immune pathways and the expansion of pro-inflammatory immune populations [109]. Altered microbial communities and increased translocation of microbial-associated molecular patterns (MAMPs) stimulate pattern-recognition receptors (PRRs), including Toll-like receptors (TLRs) and NOD-like receptors (NLRs), expressed by epithelial cells, macrophages, dendritic cells, and other immune populations [110]. Activation of these pathways promotes pro-inflammatory signaling and cytokine production, reinforcing the chronic inflammatory state associated with obesity.

Microbial metabolites further contribute to immune regulation. SCFAs, particularly butyrate and propionate, promote the differentiation and expansion of regulatory T cells through mechanisms involving histone deacetylase inhibition and G protein-coupled receptor signaling [111]. In contrast, dysbiosis-associated reductions in beneficial microbial metabolites may favor the expansion of pro-inflammatory T helper 1 (Th1) and T helper 17 (Th17) cell populations, both of which have been implicated in obesity-associated metabolic dysfunction [112].

Collectively, these findings highlight the bidirectional crosstalk between the gut microbiota and the immune system, in which alterations in microbial signals can disrupt the balance between regulatory and pro-inflammatory immune responses. This immune dysregulation provides an important mechanistic link between gut dysbiosis and the metabolic alterations associated with obesity.

### 4.5. Metabolic Consequences

Alterations in the gut microbiota have been associated with changes in adipose tissue metabolism that may contribute to its expansion and dysfunction during obesity. Experimental evidence has shown that the gut microbiota can promote body fat storage and contribute to adipose tissue development [113]. Adipose tissue dysfunction is characterized by alterations in adipogenesis, including adipocyte hypertrophy and hyperplasia, and abnormal lipid metabolism, accompanied by alterations in adipokine production and regulation [114]. As adipose tissue expands, adipocyte hypertrophy can promote local hypoxia and oxidative stress, further contributing to the production of pro-inflammatory mediators and the establishment of chronic low-grade inflammation [115,116]. In addition, gut dysbiosis may promote an inflammatory state within adipose tissue, characterized by increased macrophage infiltration and a predominance of pro-inflammatory macrophage phenotypes [117]. In turn, communication between the gut microbiota and adipose tissue is bidirectional, with adipokines acting as mediators of the gut–adipose tissue axis [118]. Taken together, these alterations impair the metabolic and endocrine functions of adipose tissue and may contribute to the development of obesity-associated metabolic complications [119].

**Figure 2:**
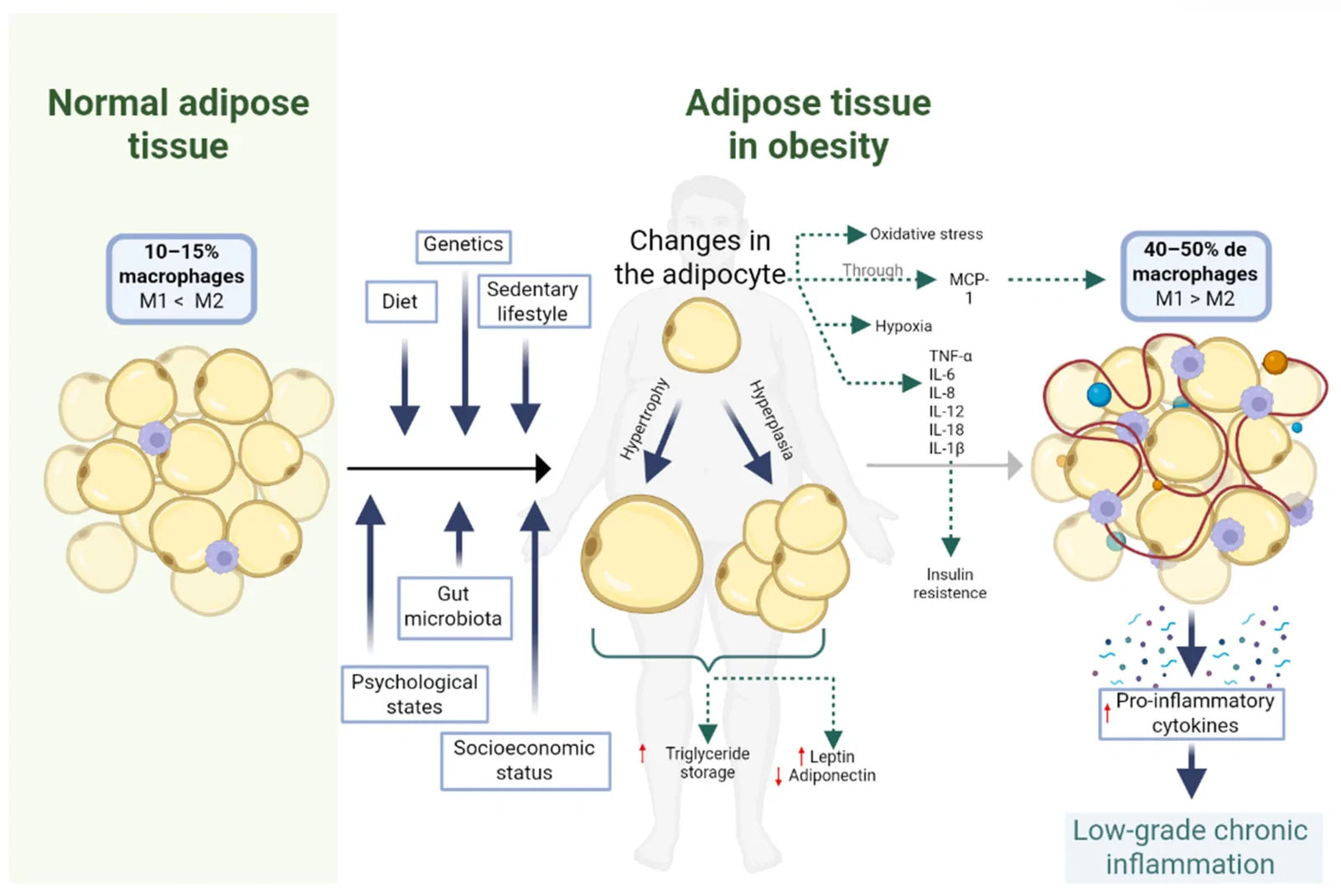
Adipose tissue dysfunction and chronic low-grade inflammation in obesity. Adipocyte hypertrophy and hyperplasia are associated with hypoxia, oxidative stress, altered adipokine production, macrophage infiltration, and increased pro-inflammatory mediators, contributing to chronic low-grade inflammation and insulin resistance. This schematic illustrates adipose tissue alterations associated with human obesity, based on evidence from human studies. The arrows indicate contributing factors and the progression of adipose tissue dysfunction, while dotted arrows illustrate associated metabolic and inflammatory pathways. The colored backgrounds distinguish normal adipose tissue from adipose tissue in obesity. MCP-1: monocyte chemoattractant protein-1; TNF-α: tumor necrosis factor-alpha; IL-1β: interleukin-1 beta; IL-6: interleukin-6; IL-8: interleukin-8; IL-12: interleukin-12; IL-18: interleukin-18; M1: classically activated macrophages; M2: alternatively activated macrophages. Created with BioRender (https://www.biorender.com; accessed on 14 August 2026).

Metabolic dysfunction associated with the gut microbiota may also extend to the regulation of insulin sensitivity through metabolic and inflammatory signals that affect tissue responses to this hormone [120]. At the molecular level, these alterations may interfere with the insulin receptor signaling pathway and its intracellular effectors, compromising metabolic responses in the liver, skeletal muscle, and adipose tissue [121]. Consequently, glucose uptake and utilization in peripheral tissues may be reduced, while the regulation of hepatic glucose production may also be impaired [122]. However, the relationship between the gut microbiota and insulin resistance is complex and likely bidirectional, and is influenced by factors such as diet, genetics, and interindividual variability [123]. Therefore, the gut microbiota should be considered one of the factors contributing to the development of obesity-associated insulin resistance rather than an isolated determinant [124]. The main obesity-associated alterations in adipose tissue and their contribution to chronic low-grade inflammation and insulin resistance are summarized in Figure 2.

In addition to its relationship with insulin sensitivity, the gut microbiota participates in other mechanisms involved in the regulation of glucose homeostasis [125]. Among these, the modulation of enteroendocrine hormones such as GLP-1 and PYY represents one of the mechanisms through which microbial activity can influence the postprandial metabolic response [126]. In addition, the gut microbiota may influence pancreatic β-cell function and the regulation of insulin secretion [127]. At the hepatic level, recent experimental evidence indicates that specific metabolites derived from microbial amino acid metabolism may contribute to the maintenance of euglycemia through the regulation of gluconeogenesis [128]. The integration of these intestinal, pancreatic, and hepatic signals positions the gut microbiota as one of the factors involved in the regulation of glucose homeostasis, and its disruption may contribute to impaired glycemic control associated with obesity [129].

## 5. Current Strategies for Microbiota Modulation

Current strategies for gut microbiota modulation include dietary interventions, supplementation with beneficial microorganisms, and more invasive approaches such as fecal microbiota transplantation (FMT) [130]. These interventions aim to modify microbial composition and activity, with potential effects on intestinal barrier function, metabolic homeostasis, and chronic low-grade inflammation associated with obesity. However, their effects can vary considerably among individuals due to differences in baseline microbiota composition, host genetics, dietary habits, and environmental exposures [131].

### 5.1. Dietary Interventions

Diet is widely regarded as one of the most accessible and influential modulators of the gut microbiota. Dietary components directly influence microbial composition, metabolic activity, and the production of bioactive metabolites that affect host physiology. Among dietary factors, fiber and polyphenols have received particular attention for their ability to promote beneficial microbial functions.

#### 5.1.1. Dietary Fiber

Because many dietary fibers resist digestion in the upper gastrointestinal tract, they become available for fermentation by microorganisms in the colon. Higher fiber consumption has been associated with changes in microbial diversity and the enrichment of bacterial groups such as *Bifidobacterium*, *Faecalibacterium*, and *Roseburia*, together with an increased representation of genes involved in carbohydrate utilization and SCFA synthesis [131].

The resulting fermentation products, particularly acetate, propionate, and butyrate, participate in processes related to epithelial barrier function, immune regulation, and metabolic homeostasis [132]. These microbial products provide a mechanistic connection between dietary fiber intake and host metabolic physiology [133]. Dietary patterns rich in fiber may promote microbiota resilience and metabolic health. However, individual responses can vary considerably due to factors such as baseline microbial composition and the capacity to ferment fiber.

#### 5.1.2. Polyphenols

Polyphenols are a diverse group of plant-derived bioactive compounds widely distributed in fruits, vegetables, tea, coffee, cocoa, nuts, and whole grains [134]. Due to their relatively low absorption in the upper gastrointestinal tract, a substantial proportion of dietary polyphenols reaches the colon, where they undergo extensive biotransformation by the gut microbiota [135]. This process generates a variety of low-molecular-weight metabolites that may exhibit greater bioavailability and distinct biological activities compared with their parent compounds [136].

The interaction between polyphenols and the gut microbiota involves reciprocal effects. On the one hand, microbial communities metabolize polyphenols into bioactive derivatives that can modulate inflammatory pathways, oxidative stress, and host metabolic responses [137]. On the other hand, polyphenols can selectively influence microbial community structure by promoting the growth of microorganisms associated with metabolic health, including *Akkermansia muciniphila* [138], *Bifidobacterium* spp., and certain *Lactobacillus* species [139], while limiting the expansion of potentially detrimental taxa. Through these interactions, polyphenols may enhance intestinal barrier integrity, modulate immune responses, and influence microbial metabolite production. Experimental studies have also shown that polyphenol-rich diets can increase microbial diversity, alter microbial functional profiles, and promote the production of metabolites associated with improved metabolic outcomes.

Clinical interventions have been reported to have beneficial effects on body weight regulation, insulin sensitivity, lipid metabolism, and systemic inflammatory markers. However, considerable interindividual variability exists, largely due to differences in gut microbiota composition and metabolic capacity [140]. Since microbial biotransformation largely determines the bioavailability and biological activity of many polyphenols, individuals may differ substantially in their capacity to generate bioactive metabolites and, consequently, in their physiological responses to supplementation. Thus, the effects of polyphenol-based interventions are influenced by complex interactions among diet, microbial communities, and host-specific factors, which may contribute to the heterogeneity of clinical outcomes [137].

### 5.2. Probiotics and Synbiotics

Probiotics are defined by their capacity to provide a health benefit when viable microorganisms are administered in sufficient quantities. Synbiotic formulations incorporate microorganisms and substrates intended to support their beneficial activity within the gastrointestinal environment. Both types of intervention have been studied for their capacity to influence intestinal microbial communities and metabolic alterations associated with obesity [141].

Several probiotic microorganisms have been investigated for their potential role in obesity management, particularly strains from the genera *Lactobacillus*, *Bifidobacterium*, and *Akkermansia* [55], as well as various of butyrate-producing bacteria. Their potential benefits have been associated with modulation of microbial community structure and function, intestinal barrier integrity, immune responses, and microbial metabolite production.

Despite encouraging findings from preclinical studies, results from human clinical trials have been considerably more variable. While some interventions have reported modest reductions in body weight, body mass index (BMI), waist circumference, inflammatory markers, and metabolic risk factors, others have failed to demonstrate significant benefits [142]. Importantly, heterogeneity across clinical trials should not be interpreted as an absence of efficacy, as meta-analyses have reported modest but statistically significant improvements in body weight, BMI, waist circumference, and other anthropometric outcomes [143].This inconsistency likely reflects the substantial heterogeneity of both host and microbiome characteristics across study populations.

One of the main challenges of probiotic therapy is the considerable functional variability among microbial strains. Even within the same species, strains can differ substantially in their metabolic activities, ability to colonize the gastrointestinal tract, capacity to modulate host immune responses, and interactions with the existing microbial community. In addition, growing evidence suggests that many probiotic strains do not readily integrate into the resident microbiota and frequently show limited long-term persistence after administration. Consequently, the effects of probiotic supplementation are often temporary and may depend largely on the composition and ecological characteristics of the host’s pre-existing gut microbiome [142].

Synbiotics have been proposed to enhance probiotic efficacy by providing complementary substrates that support microbial survival and activity. Although several studies suggest that synbiotics may produce greater metabolic benefits than probiotics alone, findings remain heterogeneous due to differences in strain selection, substrate composition, dosage, treatment duration, and study design [130]. Moreover, the ecological complexity of the gut microbiome makes it difficult to predict how administered microorganisms will interact with existing microbial communities.

Taken together, current findings indicate that probiotic and synbiotic therapies do not produce consistent outcomes in every individual. Rather, their efficacy is shaped by the dynamic interplay between host factors and microbial communities. Differences in baseline gut microbiota composition and functional capacity may contribute to distinct responder and non-responder phenotypes, suggesting that microbiome-based stratification could help identify individuals more likely to benefit from these interventions [144]. These factors limit the predictability and reproducibility of their effects across individuals.

### 5.3. Prebiotics

Prebiotics are substrates that are selectively utilized by host microorganisms and confer health benefits to the host. Commonly studied prebiotics include inulin, fructooligosaccharides, galactooligosaccharides, resistant starches, and various non-digestible polysaccharides. By serving as fermentable substrates for specific microbial populations, prebiotics aim to promote beneficial microbial functions and improve host metabolic health [133].

Prebiotic supplementation has been shown to increase the abundance of microorganisms associated with metabolic health, including *Bifidobacterium* spp., *Akkermansia muciniphila*, and several SCFA-producing bacteria [145]. Through microbial fermentation, prebiotics stimulate the production of acetate, propionate, and butyrate, thereby influencing microbial functions involved in intestinal and metabolic homeostasis [146]. These changes have been associated with improvements in several obesity-related metabolic outcomes.

Despite these promising effects, the selectivity of prebiotics remains an important challenge. Early concepts assumed that specific substrates would selectively stimulate a limited number of beneficial microorganisms. However, advances in metagenomic and metabolomic analyses have revealed that prebiotics frequently affect complex microbial networks rather than individual taxa [147]. Therefore, microbial responses are often broader and less predictable than originally anticipated.

A further limitation is the substantial interindividual variability in treatment response. The metabolic effects of prebiotics depend heavily on the recipient’s baseline microbiome composition, microbial gene repertoire, dietary habits, and host physiology. As a result, identical prebiotic interventions may generate markedly different microbial and metabolic outcomes across individuals [148]. In addition, although prebiotics remain among the safest and most accessible microbiota-modulating interventions, their relatively indirect mechanism of action and limited microbial selectivity may restrict their ability to achieve precise microbiome remodeling. Therefore, despite their potential benefits, the effects of prebiotic interventions remain dependent on complex ecological interactions within the gut microbial community.

### 5.4. Antibiotics

Antibiotics were among the first tools used to intentionally modify the gut microbiota. Their administration can substantially reduce bacterial populations and reshape microbial community structure, leading to significant changes in microbial metabolism and host physiological processes. These effects have also provided experimental evidence of the contribution of intestinal microorganisms to host metabolic regulation [149].

In some animal models, antibiotic administration has been associated with reduced adipose tissue inflammation, improved insulin sensitivity, and protection against diet-induced metabolic dysfunction [149,150]. The direction and magnitude of these metabolic effects may vary according to the experimental model, antibiotic regimen, and timing of exposure, particularly during critical periods of microbiota development [151]. These findings initially suggested that manipulation of microbial communities could represent a viable strategy for treating obesity and related metabolic disorders. However, such benefits are often transient and accompanied by widespread disruption of microbial ecosystem structure and function.

Despite their ability to modify the gut microbiota, the limited taxonomic selectivity of many antibiotics represents a major obstacle to their use as precision microbiome interventions. Broad-spectrum agents can affect numerous bacterial populations simultaneously, including microorganisms that perform beneficial functions within the intestinal ecosystem. Such disturbances may diminish microbial diversity, weaken colonization resistance, and interfere with metabolic activities maintained by resident bacteria [149]. The resulting ecological changes can facilitate the expansion of opportunistic pathogens, while restoration of the original microbial community may be prolonged or incomplete. Consequently, antibiotic exposure can leave persistent alterations in microbiota composition even after treatment has ended.

Evidence from both experimental and epidemiological studies further indicates that repeated antibiotic exposure may have adverse metabolic consequences. Early-life antibiotic administration has been associated with alterations in microbiota development, increased adiposity, and elevated risk of obesity later in life [152]. Similar findings in animal models suggest that antibiotic-induced perturbations during critical developmental windows may have long-lasting effects on host metabolism and immune maturation.

In addition to their ecological limitations, the widespread use of antibiotics contributes to the emergence and dissemination of antimicrobial resistance. This situation represents a major global public health concern. Therefore, although antibiotics have provided valuable insights into microbiota–host interactions, their broad and non-selective effects make them poorly suited for long-term microbiome engineering. Together, these ecological and clinical limitations restrict their applicability as a controlled strategy for long-term gut microbiota modulation.

### 5.5. Fecal Microbiota Transplantation

FMT seeks to reconstitute intestinal microbial communities by introducing donor-derived microorganisms into a recipient. Its established effectiveness against recurrent *Clostridioides difficile* infection has encouraged investigations into whether this approach could also benefit individuals with obesity or metabolic disorders [153]. Experimental findings support a role for transferred microbial communities in shaping host metabolic phenotypes: microbiota originating from lean donors have been associated with improvements in insulin sensitivity, whereas microbiota from donors with obesity may contribute to increased adiposity and metabolic impairment [154].

Nevertheless, clinical reports on obesity have been less consistent. Although some studies have reported short-term improvements in insulin sensitivity and microbial diversity, long-term effects on body weight and metabolic health remain modest and variable [155]. This variability is influenced by factors such as donor characteristics, recipient microbiome composition, engraftment efficiency, diet, and host immune responses. In addition, safety concerns regarding the transfer of pathogenic microorganisms, antimicrobial resistance genes, and other poorly characterized biological components continue to limit the widespread clinical application [156]. Thus, although FMT remains one of the most effective methods for reshaping the gut microbiome, its complexity, variability, and limited precision can restrict the predictability and control of microbiome remodeling.

## 6. Precision Microbiome Modulation: Emerging Concepts

As described above, conventional strategies for gut microbiota modulation can modify the composition and activity of microbial communities, but responses to these interventions vary considerably among individuals [157,158]. This variability may be influenced by factors such as baseline microbiota composition, health status, dietary patterns, and intervention-specific characteristics [159]. In addition, microbiome interventions may produce effects that extend beyond the initially targeted component due to ecological interactions within the microbial community [160]. These limitations have driven the development of strategies aimed at selectively targeting defined components of the microbiome [161].

In this context, precision microbiome modulation involves the development of interventions capable of selectively acting on defined components of the gut ecosystem [162]. These strategies can target microorganisms (microbiota), functions, or microbial products previously identified as relevant to a specific phenotype [163]. The development of technologies with greater taxonomic and functional resolution, together with advances in microbiome engineering, has expanded the ability to identify these targets and design specific interventions [164]. In this way, precision modulation seeks to target specific components of the microbiome while limiting unnecessary alterations to the remaining microbial community [165].

Among the biological tools developed for this purpose are genetically engineered bacteria, whose behavior can be programmed using synthetic biology approaches to perform defined functions within the gut environment. These modifications can improve characteristics such as survival and resistance to stressful conditions, while also introducing or enhancing therapeutic functions [166]. Moreover, these microorganisms can be engineered to detect specific signals within the gut microenvironment and respond through the in situ production or release of biologically active molecules [167]. This capability has led to the development of programmable microbial systems that combine sensing and response functions, enabling localized action in response to specific conditions within the gut environment [168].

These tools can operate through distinct mechanisms. While some approaches aim to introduce or modify functions using engineered microorganisms, others are designed to selectively eliminate bacterial populations or modify specific characteristics of resident microorganisms [169]. This diversity of tools enables interventions to be tailored according to the nature of the identified target, rather than seeking broad modification of the microbial community [170].

Among strategies aimed at the selective elimination of bacterial populations, bacteriophages represent one of the most promising biological tools due to their specificity for particular bacterial hosts [171]. This property provides the opportunity to target specific microorganisms while largely preserving other members of complex microbial communities [172]. Therefore, bacteriophages represent a particularly relevant tool for targeted microbiome modulation by providing an intervention strategy based on the selective targeting of bacterial populations [173].

## 7. Bacteriophage Therapy as a Targeted Modulation Strategy

### 7.1. Basics of Phage Biology Relevant to Microbiota Modulation

Bacteriophages, or phages, are bacterial viruses that have been investigated for more than a century and have attracted renewed therapeutic interest as alternatives to conventional antimicrobial agents [174]. Many phages investigated for therapeutic use belong to *Caudoviricetes*, a class comprising double-stranded DNA viruses with a protein capsid and a tail structure [175,176]. Historically, tailed phages have been grouped according to tail morphology into myoviruses, characterized by contractile tails; siphoviruses, with long and flexible tails; and podoviruses, which possess short tails [177].

A defining feature of phages is their narrow host range, which is largely determined by the specificity of their receptor-binding proteins (RBPs) toward distinct receptors on the bacterial cell surface [178]. This high specificity enables the selective targeting of defined bacterial populations, making phages particularly attractive for applications requiring precise modulation of complex microbial communities, such as the gut microbiota.

Upon infection of a susceptible host, phages can follow, mainly, two life cycles: lytic or lysogenic. Temperate phages, which can establish a lysogenic cycle, integrate their genome into the bacterial chromosome, forming a prophage [175]. This state allows the phage to persist within the host, replicating passively alongside the bacterial genome. Under adverse environmental conditions, such as thermal stress, nutrient deprivation, or antibiotic exposure, prophages may be induced to enter the lytic cycle [179]. However, because the lysogenic lifestyle prioritizes genome maintenance over immediate host lysis, temperate phages are generally considered unsuitable for therapeutic applications.

In contrast, virulent phages, which exclusively undergo the lytic cycle, hijack the host’s metabolic machinery to replicate their genomes, assemble new virions, and ultimately lyse the bacterial cell, releasing progeny phages [180]. This strictly lytic behavior is a critical requirement for phage therapy and targeted microbiota modulation.

Therefore, careful evaluation of candidate phages is essential prior to therapeutic application. Beyond confirming a strictly lytic lifestyle, often achieved through genomic and bioinformatic analyses, phages must be screened to ensure the absence of genes associated with bacterial virulence, antibiotic resistance, or lysogeny [181]. These elements may be acquired during previous infection cycles and, if transferred to bacterial hosts, could inadvertently enhance bacterial fitness or persistence [182]. Given that bacteria possess diverse defense mechanisms against phage infection, including systems that can inactivate phages and potentially retain exogenous genetic material, the use of inadequately characterized phages may counteract the intended therapeutic effect [183].

### 7.2. Advantages over Conventional Approaches

The primary feature that makes bacteriophages an attractive alternative for targeted microbiota modulation is their high specificity. In most cases, the host range of a given phage is restricted to a limited number of strains within a particular bacterial species, although broader host ranges spanning multiple genera have occasionally been reported [184]. These interactions are governed by the recognition of bacterial surface receptors by phage RBPs, meaning that the presence and accessibility of these receptors ultimately determine a phage’s infectivity [178].

This specificity becomes particularly advantageous in complex microbial ecosystems such as the gut microbiota, where a high diversity of microorganisms performs essential and often interdependent functions [185]. Conventional broad-spectrum antimicrobials can disrupt these communities, leading to dysbiosis and enabling the expansion of resilient or opportunistic taxa that occupy newly available ecological niches [186]. In contrast, targeted approaches such as phage therapy allow for the selective removal of specific bacterial species or strains associated with pathological conditions, such as inflammatory bowel disease, while preserving the core microbiota and maintaining a state of eubiosis [187].

The capacity of lytic phages to multiply in susceptible bacteria is another characteristic of therapeutic interest. Following successful infection, bacterial replication machinery is redirected toward phage production, potentially yielding tens to hundreds of viral particles per infected cell, with reported burst sizes commonly ranging from approximately 50 to 200 [188]. After bacterial lysis, released progeny may infect additional susceptible cells, allowing phage populations to expand locally when suitable hosts remain available. This process can increase phage exposure at the bacterial target, although its magnitude depends on host availability and environmental conditions. As susceptible bacterial populations decline, opportunities for further phage replication also decrease [189].

There is also emerging evidence suggesting that phages may interact with eukaryotic cells, including internalization and potential degradation within cellular compartments [190]. While the physiological relevance of these interactions remains under investigation, they may contribute to phage clearance and further support their transient and self-limiting behavior within the host.

### 7.3. Phage–Microbiota–Host Interactions

The gut microbiota plays a fundamental role in human health, influencing processes such as immune system regulation, vitamin production, and protection against invading pathogens [191]. Moreover, the diversity and abundance of the microorganisms that comprise this ecosystem (including bacteria, archaea, fungi, protozoa, and viruses) are frequently associated with the onset and progression of multiple diseases [192]. Consequently, the targeted modulation of specific microbial groups has emerged as an area of growing interest for addressing gut microbiota dysbiosis, with bacteriophages standing out as one of the most promising tools for precision microbiota modulation [193].

However, the interaction between phages, the microbiota, and the human host represents a highly complex tripartite system. Although substantial progress has been made in characterizing dual interactions, particularly those between phages and bacteria, understanding these dynamics within the context of a complex ecosystem remains a major challenge.

Bacteria constitute the dominant component of the gut microbiota, with six major phyla commonly identified: Bacillota, Bacteroidota, Actinomycetota, Pseudomonadota, Fusobacteriota, and Verrucomicrobiota [185]. Among these, Bacillota and Bacteroidota are generally the most abundant phyla in healthy individuals. Advances in high-throughput sequencing and metagenomic approaches now provide substantially greater taxonomic resolution, enabling the characterization of microbial communities at the species and even strain level [187]. This has facilitated the identification of specific bacterial taxa associated with either health or disease states. For example, genera such as *Bacteroides* and *Prevotella* have been proposed as markers of a healthy adult microbiota [194], whereas *Klebsiella pneumoniae* has been associated with inflammatory bowel disease [187]. Similarly, *Enterobacter cloacae* has been linked to chronic inflammation and lipid accumulation [195], highlighting the role of specific bacterial populations in metabolic disorders such as obesity.

Despite bacteria constituting the largest fraction of the gut microbiota, viruses also represent a significant component of this ecosystem, with bacteriophages making up the vast majority of these viral populations [196]. Collectively referred to as the human gut virome, these viral communities comprise bacteriophages, viruses infecting eukaryotic hosts, and a substantial proportion of viral sequences that remain taxonomically unclassified or functionally uncharacterized [197]. This poorly characterized fraction, often referred to as viral dark matter, reflects important limitations in current viral reference databases and highlights the considerable diversity of the intestinal virome that remains unexplored [198]. Hence, the composition, diversity, and dynamics of bacterial communities are strongly influenced by phage activity. Interestingly, the order *Crassvirales* has been identified as one of the most widespread viral groups in the human gut, with a prevalence reaching up to 73% and relative abundances approaching 99% in some individuals [199]. This order comprises tailed double-stranded DNA phages belonging to the class *Caudoviricetes*, whose hosts are primarily members of the phylum Bacteroidota, particularly abundant anaerobic bacteria that play key roles in maintaining gut homeostasis [200].

Given the remarkable abundance and persistence of both *Crassvirales* phages and Bacteroidota populations in the human gut, their interactions likely represent one of the dominant ecological forces shaping the structure and function of intestinal microbial communities. More broadly, these observations highlight the fundamental role of phages in regulating bacterial populations and influencing ecosystem stability within the gut [201]. However, when considering bacteriophages as therapeutic tools for microbiota modulation, it is essential to carefully identify the microbial targets of interest and predict the ecological consequences of phage intervention [193]. The objective should not be indiscriminate bacterial depletion, but rather the selective removal of disease-associated taxa while preserving beneficial microbial functions and maintaining a state of eubiosis [187].

Although substantial progress has been made in identifying associations between the gut microbiota and obesity, establishing direct causal relationships between individual microorganisms and disease phenotypes remains challenging [202]. Obesity is a multifactorial condition characterized by complex interactions among diet, host genetics, immune responses, and microbial ecology, making it unlikely that a single microorganism is solely responsible for disease onset or progression [203]. Nevertheless, several bacterial taxa have been consistently associated with metabolic health or dysfunction, particularly through their ability to modulate chronic low-grade inflammation, intestinal barrier integrity, endotoxemia, and host metabolic signaling, as summarized in Table 2. For example, *Enterobacter cloacae* has been implicated in promoting inflammation and metabolic endotoxemia, whereas *Akkermansia muciniphila*, *Faecalibacterium prausnitzii*, *Christensenella minuta*, and *Parabacteroides distasonis* have been associated with anti-inflammatory effects and improved metabolic outcomes (Table 2). These findings suggest that therapeutic interventions should move beyond broad taxonomic descriptions and focus on identifying functionally relevant microbial targets. Future efforts should therefore prioritize strain-level and genome-resolved characterization of obesity-associated microorganisms to uncover the specific genetic determinants driving pathogenic or protective phenotypes, as has already been explored in other microbiota-associated disorders, such as inflammatory bowel disease [187]. Likewise, a deeper understanding of phage–bacteria–host interactions will be essential to predict the ecological and physiological consequences of targeted phage interventions [204]. The most relevant studies investigating phage-mediated modulation of the gut microbiota are summarized in Table 3. Together, these studies demonstrate that bacteriophages can selectively reduce target bacterial populations while exerting broader effects on microbial community structure, metabolite production, intestinal inflammation, and host physiology. Importantly, several investigations have shown that phage administration can increase the abundance of beneficial taxa, promote short-chain fatty acid production, improve gut barrier function, and reduce inflammatory markers without causing the widespread disruption typically associated with broad-spectrum antibiotics. These findings support the concept that phages can function as ecological regulators capable of reshaping microbial communities in a controlled manner rather than simply eliminating bacterial hosts.

Nevertheless, the current body of evidence also reveals important limitations. Available investigations have largely relied on simplified microbial systems, animal experiments, and ex vivo models, leaving substantial uncertainty about whether their findings can be reproduced in humans. Furthermore, the long-term ecological effects of phage exposure, the selection of resistant bacterial populations, and the consequences of eliminating particular strains from microbiomes that differ substantially between individuals have not been adequately established [205]. Notably, only a limited number of studies have focused on obesity-associated microorganisms, despite growing evidence implicating specific bacterial strains in chronic low-grade inflammation, metabolic endotoxemia, and altered energy metabolism [206]. Future research should therefore integrate genome-resolved microbiome analyses, strain-level target identification, and mechanistic characterization of phage–bacteria–host interactions to enable the rational design of precision phage therapies [205]. Such efforts will be critical for identifying microbial targets with causal roles in metabolic disease and for developing interventions capable of selectively eliminating detrimental bacterial populations while preserving beneficial microbial functions and maintaining gut ecosystem stability [207].

**Table 3:** Experimental studies evaluating bacteriophage-mediated modulation of gut microbiota composition, function, and host metabolic or inflammatory outcomes.

Bacteriophages	Target Bacteria	Study Objective and Study Type	Main Findings	Ref.
HKP09	*Bacteroides uniformis* DSM 6597	To evaluate how phage predation affects gut microbial community structure and metabolism in a defined microbiota. Study type: In vitro synthetic-community model and gnotobiotic mouse study	HKP09 induced a transient reduction in *B. uniformis*, altered the abundance of non-target community members, and modified fecal amino acid and fermentation metabolite profiles, demonstrating that phage-mediated perturbations can reshape microbiome function beyond the targeted host.	[208]
T4, F1, B40-8, VD13	*Escherichia coli*, *Clostridium sporogenes*, *Bacteroides fragilis*, *Enterococcus faecalis*	To determine how targeted phage predation influences gut microbiota dynamics and metabolite production. Study type: In vitro and gnotobiotic mouse study	Phages selectively reduced susceptible bacteria, triggered cascading effects on non-target taxa, and altered gut metabolite profiles, demonstrating that phages can be used to modulate microbiota composition and function.	[204]
PreforPro cocktail (LH01, LL5, T4D, LL12)	*Escherichia coli*	To assess the effects of oral phage supplementation on gut microbiota and inflammatory markers in healthy adults. Study type: Human randomized, double-blind, placebo-controlled crossover trial.	Reduced fecal *E. coli* without disrupting overall microbiota diversity, increased beneficial butyrate-producing taxa (*Eubacterium* spp.), decreased taxa related to *Clostridium perfringens*, and reduced circulating IL-4 levels.	[193]
PreforPro cocktail + *Bifidobacterium animalis* subsp. *lactis* BL04	*Escherichia coli*	To determine whether phage supplementation enhances probiotic-mediated gut health benefits. Study type: Human randomized, double-blind, placebo-controlled, parallel-arm trial.	Increased *Lactobacillus* and SCFA-producing bacteria, improved gastrointestinal inflammation scores, and enhanced the effects of the probiotic without causing dysbiosis.	[209]
Intesti bacteriophage cocktail	Primarily Enterobacteriaceae including *Escherichia coli*	To evaluate the impact of a commercial phage cocktail on healthy human gut microbiota. Study type: In vitro human colon fermentation model.	Reduced *E. coli* abundance while producing minimal alterations in bacterial community composition and virome structure, supporting the specificity and safety of phage-based microbiota modulation.	[196]
AS5 + AS6	*Enterobacter cloacae*	To evaluate whether a phage cocktail can selectively eliminate an obesity-associated bacterium in an ex vivo colon model. Study type: Ex vivo colon fermentation model.	Selectively reduced *E. cloacae* while preserving overall microbiota composition, altered beta diversity, and increased lactic and butyric acid concentrations in specific colonic regions.	[210]
Human fecal virome (VLP pool)	Multiple gut bacteria	To investigate phage-host dynamics in a defined gut microbial community. Study type: In vivo gnotobiotic mouse model.	Phage transfer caused reproducible reductions in specific bacterial taxa, demonstrated community-wide phage-host interactions, and showed that phages can shape microbial ecology in vivo.	[211]
Five-phage cocktail (BiomX consortium)	*Klebsiella pneumoniae*	To selectively suppress IBD-associated *K. pneumoniae* strains and reduce intestinal inflammation. Study type: Preclinical animal studies and human safety study.	Efficiently reduced *K. pneumoniae* colonization, attenuated intestinal inflammation and disease severity in murine models, and demonstrated safety and viability in healthy human volunteers.	[187]
Salmonella-targeting phage cocktail encapsulated in hydrogel microspheres	*Salmonella enterica* serovar Typhimurium	To develop a microbiota-editing platform that improves phage delivery and therapeutic efficacy. Study type: Murine *Salmonella* colitis model.	Reduced intestinal pathogen burden by ~2000-fold, decreased TNF-α, IL-6, and IL-1β levels, restored gut homeostasis, and avoided antibiotic-associated microbiota disruption.	[212]
MS2 + P22 phage cocktail	Gut microbiota associated with T2D dysbiosis	To evaluate whether phages can alleviate type 2 diabetes through microbiota modulation. Study type: In vivo mouse model of type 2 diabetes.	Increased SCFA-producing taxa (*Blautia*, *Romboutsia*), reduced opportunistic pathogens, lowered LPS and pro-inflammatory cytokines, improved gut barrier function, and attenuated diabetic symptoms.	[213]

IBD: inflammatory bowel disease; T2D: type 2 diabetes; VLP: virus-like particle.

## 8. Challenges and Limitations of Phage-Based Modulation

Despite the numerous advantages that bacteriophages offer over conventional microbiota-targeting interventions, several challenges and limitations must be considered before their widespread implementation. Among these challenges, the emergence of phage-resistant bacterial variants is particularly relevant because bacterial populations can develop multiple defenses against viral infection [175]. These defenses include superinfection exclusion, CRISPR-Cas-mediated immunity, abortive infection, and restriction–modification systems [214]. However, the most commonly reported mechanism in therapeutic settings is receptor modification or loss [215].

This mechanism occurs when spontaneous mutations alter or eliminate the bacterial surface receptor recognized by a phage. As a result, resistant mutants gain a selective advantage under phage predation and can become dominant within the bacterial population. Importantly, receptor-mediated phage resistance is often associated with physiological trade-offs that depend on the biological function of the affected receptor [215]. For example, mutations affecting flagella or pili may reduce bacterial motility, whereas modifications of cell envelope components can increase membrane permeability and restore susceptibility to antibiotics [216]. Similarly, alterations in capsular polysaccharides may impair adhesion, colonization, or biofilm formation [217]. Consequently, although phage-resistant bacteria may survive treatment, they frequently exhibit attenuated fitness or virulence, suggesting that the emergence of resistance does not necessarily imply therapeutic failure. In some cases, phage-driven evolutionary trade-offs may even enhance treatment outcomes by reducing the pathogenic potential of the target population [218].

Another factor that can influence phage efficacy is their stability under environmental stress conditions, particularly exposure to acidic pH. To exert a microbiota-modulating effect, orally administered phages must reach the appropriate region of the gastrointestinal tract at sufficient titers [181]. This requires surviving passage through the stomach, one of the most hostile environments in the human body, characterized by proteolytic enzymes and a pH that can range from approximately 1 to 3. Numerous studies have demonstrated that many phages rapidly lose infectivity when exposed to pH values below 2, highlighting the need for effective delivery systems [219].

Microencapsulation has emerged as the most extensively studied strategy for protecting phages during gastrointestinal transit. A wide variety of biocompatible matrices have been investigated, including alginate, chitosan, whey protein, gelatin, carrageenan, pectin, cellulose derivatives, and synthetic polymers [220]. These formulations are typically designed to remain stable under gastric conditions while enabling controlled phage release in the intestine through pH-dependent dissolution or enzymatic degradation [191]. In addition to improving survival under acidic conditions, microencapsulation can enhance storage stability, protect phages from environmental stresses, and facilitate the administration of phage cocktails. Several studies have reported substantial improvements in phage recovery following simulated gastrointestinal transit, supporting the potential of encapsulation technologies for clinical and nutritional applications [181,220]. Alternatively, some studies have addressed gastric inactivation through the administration of antacids prior to phage treatment, demonstrating successful recovery of viable phages in fecal samples [187]. Although considerably simpler than encapsulation-based approaches, the potential benefits and limitations of antacid administration should be carefully evaluated according to the intended therapeutic application.

Phage selection and biosafety also represent critical considerations for microbiota modulation strategies. From a practical perspective, therapeutic phages should possess a well-defined host range, ideally restricted to the bacterial strain or species targeted for elimination to minimize unintended disturbances to the gut microbiota [187]. Furthermore, candidate phages should demonstrate robust suppressive activity against their intended targets in both in vitro and in vivo models [216]. Historically, strictly lytic phages have been considered the gold standard for therapeutic applications because of concerns associated with lysogeny and horizontal gene transfer. More recently, however, some researchers have argued that the key safety criterion may not necessarily be the inability to establish lysogeny, but rather the inability to mediate the transfer of undesirable genetic material between bacterial hosts [221]. This evolving debate will likely shape future regulatory and scientific frameworks for phage therapy. Nevertheless, the prevailing approach to therapeutic phage selection emphasizes excluding candidates that carry genes encoding antibiotic resistance, virulence determinants, or functions associated with lysogeny [181]. This precaution is especially relevant in the intestine, where the high density and taxonomic diversity of bacterial communities create conditions that may facilitate horizontal gene exchange [182]. The inadvertent dissemination of resistance or virulence determinants could not only compromise treatment efficacy but also enhance the persistence, adaptability, or pathogenicity of microbial populations within the intestinal environment.

Finally, regulatory considerations remain an important challenge for the broader implementation of phage-based microbiota modulation [181]. Unlike clinical phage therapy, which is generally regulated as a medicinal product, most commercially available phage formulations intended to modulate the gut microbiota are currently marketed as dietary supplements, allowing them to enter the market through less stringent regulatory pathways [193,222]. This classification has facilitated innovation and commercialization, and several phage preparations have been recognized as Generally Recognized as Safe (GRAS) by the U.S. Food and Drug Administration (FDA) since 2006. More recently, bacteriophages have been proposed as a new generation of “precision prebiotics” because of their ability to selectively reduce specific bacterial populations while indirectly promoting the growth of beneficial microorganisms [201,223]. Although this concept remains under discussion and has not yet been formally incorporated into regulatory frameworks, it reflects a growing recognition of phages as ecological modulators rather than conventional antimicrobials.

Nevertheless, the relatively permissive regulatory environment governing dietary supplements may result in substantial variability in product quality, characterization, and clinical validation [222]. Given the increasing interest in phage-based microbiota interventions, regulatory agencies may eventually need to establish specific guidelines addressing genomic characterization, manufacturing consistency, formulation stability, safety assessment, and demonstration of biological efficacy [224]. Regardless of their regulatory classification, phage-based products should be supported by rigorous preclinical and clinical evidence to ensure both safety and effectiveness [187]. Such standards would not only provide greater protection for consumers but also help maintain scientific credibility and public confidence in phage-based technologies, thereby facilitating their long-term integration into precision nutrition and microbiome-targeted healthcare strategies.

## 9. Engineering Next-Generation Phage-Based Interventions

### 9.1. Rational Design of Phage Cocktails

Combining bacteriophages with complementary biological properties offers a strategy to address two limitations of individual phages: bacterial resistance and restricted host range [225]. The optimal composition of a phage cocktail should ultimately be dictated by the therapeutic target and the microbial ecology underlying the disease of interest [226].

When a specific bacterial strain has been demonstrated to play a causal role in disease development, phage cocktail design should focus on combining phages that recognize different bacterial surface receptors [215]. Under this approach, spontaneous receptor modifications that confer resistance to one phage are unlikely to prevent infection by the remaining members of the cocktail [225]. Moreover, the accumulation of multiple receptor mutations often imposes substantial physiological costs on the bacterium, including reduced motility, impaired biofilm formation, decreased colonization capacity, or increased susceptibility to antibiotics [216]. Consequently, even if resistant variants emerge, their reduced fitness may limit their persistence within the microbial community and contribute to the overall therapeutic efficacy of the cocktail.

Although this represents the ideal scenario, obesity and other metabolic disorders are unlikely to be driven by a single bacterial strain. Instead, current evidence suggests that disease-associated phenotypes result from complex microbial consortia composed of multiple species or genera that collectively promote chronic low-grade inflammation, metabolic endotoxemia, or altered metabolite production [202,206]. Under these circumstances, phage cocktails should be designed to maximize coverage of the relevant pathogenic bacterial populations rather than focusing on a single target. This can be achieved by combining phages with complementary host ranges, thereby increasing the probability that at least one phage will effectively target the disease-associated bacteria present in a given individual’s microbiota [225,226]. Importantly, each component of the cocktail should demonstrate strong lytic activity against its intended target, ensuring that bacterial suppression is sufficient to induce measurable ecological and physiological changes [227].

As genome-resolved microbiome studies continue to identify bacterial strains causally associated with obesity and metabolic dysfunction, rationally designed phage cocktails may become increasingly effective adjunctive strategies for precision nutrition [228]. Rather than indiscriminately altering the gut microbiota, these formulations could selectively eliminate pro-inflammatory bacterial populations while preserving beneficial microbial communities and their metabolic functions [187,193].

### 9.2. Combination Therapies

Although selective bacterial depletion represents the primary mechanism underlying phage therapy, combining phages with other microbiota-directed interventions may further enhance therapeutic efficacy [209]. The ecological niches created following phage-mediated reduction in disease-associated bacteria provide an opportunity for beneficial microorganisms to expand, potentially accelerating the restoration of a healthy microbial ecosystem [204]. Consequently, combination therapies are likely to represent the next generation of microbiota-targeted interventions.

One promising strategy involves combining phage cocktails with probiotics containing bacterial species that have demonstrated beneficial effects in obesity and metabolic disorders. Among the microorganisms investigated for their metabolic relevance, *Akkermansia muciniphila* has been linked to intestinal barrier maintenance, lower metabolic endotoxemia, improved insulin responsiveness, and attenuation of adipose tissue inflammation [53,54,55]. Other extensively studied genera, including *Bifidobacterium* and *Lactobacillus*, have been associated with improvements in epithelial permeability, circulating LPS levels, immune regulation, and microbial production of short-chain fatty acids [229]. Emerging next-generation probiotics, including *Christensenella minuta*, *Faecalibacterium prausnitzii*, *Blautia wexlerae*, *Parabacteroides distasonis*, and *Roseburia intestinalis*, have also demonstrated promising anti-inflammatory and metabolic effects through mechanisms such as butyrate production, bile acid metabolism, maintenance of epithelial barrier integrity, modulation of regulatory T-cell responses, and inhibition of pro-inflammatory cytokine production [56,57,58,59,61,62].

Although the co-administration of prebiotics represents an attractive complementary strategy for phage-based microbiota modulation, experimental evidence supporting this approach remains remarkably limited. Most studies have evaluated bacteriophages either as standalone interventions [204] or in combination with probiotics [209], whereas investigations combining phages with classical prebiotics, such as inulin, fructooligosaccharides (FOS), galactooligosaccharides (GOS), resistant starch, β-glucans, or arabinoxylans [230], are scarce. Nevertheless, such combinations have a sound ecological rationale. Whereas bacteriophages can selectively reduce disease-associated bacterial populations, prebiotics provide substrates that are selectively utilized by beneficial members of the resident microbiota, thereby promoting their growth and metabolic activity [201,231]. In principle, this complementary strategy could facilitate the occupation of ecological niches generated by phage-mediated bacterial depletion, thereby accelerating microbial community recovery, enhancing the production of beneficial metabolites such as short-chain fatty acids, and contributing to the restoration of gut ecosystem function [187,201,204]. However, whether these theoretical advantages translate into synergistic effects on microbiota remodeling, metabolic homeostasis, and host health remains to be systematically investigated.

## 10. Future Perspectives and Clinical Translation

The future of phage-based microbiota modulation is both promising and challenging. The body of evidence generated thus far supports the potential of bacteriophages as tools for precision microbiome engineering, with multiple studies demonstrating their ability to selectively target specific bacterial populations while preserving overall microbial community structure and function [201,204]. Nevertheless, the available evidence remains insufficient for broad clinical implementation, making rigorous preclinical evaluation and appropriately designed human trials essential next steps. A critical aspect of this process will be the rational selection of bacterial targets based on demonstrated causal relationships with disease rather than simple associations derived from microbiome profiling studies [63,64,187].

One of the major challenges facing the clinical translation of phage-based interventions for obesity is the identification of broadly applicable therapeutic targets. Unlike infectious diseases, where a single pathogen is often responsible for disease manifestation, obesity is a multifactorial disorder characterized by substantial interindividual variability in microbiota composition, dietary habits, host genetics, and metabolic responses [202]. Consequently, the development of universally applicable phage formulations may prove difficult. While personalized phage therapy has already demonstrated success in clinical settings, particularly for refractory bacterial infections, the individualized nature of these treatments substantially increases production costs and logistical complexity [232]. Scaling up these approaches would also require standardized manufacturing, consistent phage potency and formulation stability, and dosing regimens that maintain effective intestinal exposure. These requirements may further increase costs, particularly for individualized formulations compared with broadly applicable phage cocktails [233]. In the context of obesity and other metabolic disorders, where phages are more likely to function as modulators of disease-associated microbial populations rather than life-saving interventions, economically sustainable strategies will be required. Therefore, future formulations will likely need to target bacterial strains or functional groups that are consistently associated with disease across defined patient subpopulations rather than attempting to address the entire spectrum of obesity-related dysbiosis [64,187].

Achieving this goal will require large-scale screening efforts integrating metagenomics, culturomics, comparative genomics, metabolomics, and host phenotypic data to identify microbial targets with genuine mechanistic relevance [228]. For obesity-related phage interventions, target prioritization should integrate reproducible strain-level associations with disease, mechanistic evidence, and the relationship between bacterial characteristics and patients’ metabolic and inflammatory profiles [234]. A strain should only be considered a sufficiently validated preclinical target when its causal contribution to metabolic dysfunction has been demonstrated in relevant animal models, measurable metabolic or inflammatory benefits follow its suppression, and selective phage-mediated depletion is feasible without substantial disruption of beneficial microbial populations. These criteria provide a framework for target selection rather than evidence of established clinical efficacy [235]. Candidate bacteria should then be evaluated through a rigorous translational pipeline that includes in vitro, ex vivo, and in vivo validation, followed by controlled clinical trials. The workflow proposed by Federici et al. [187] provides an excellent framework for the systematic identification and validation of microbiota-based therapeutic targets and could serve as a model for future phage development programs.

Beyond target identification, a deeper understanding of phage–bacteria–host interactions remain essential. While phages are generally viewed as highly specific biological agents, their effects frequently extend beyond the targeted bacterial host through ecological interactions, metabolic cross-feeding networks, and immune-mediated mechanisms [201]. Furthermore, the consequences of bacterial lysis, including the release of microbial metabolites, cell wall components, endotoxins, and other immunologically active molecules, remain incompletely understood in the context of chronic metabolic diseases [205]. Elucidating these complex interactions will be critical for predicting therapeutic outcomes, identifying potential adverse effects, and optimizing treatment strategies.

Ultimately, the successful implementation of these strategies could position bacteriophages as one of the most powerful tools in precision nutrition and microbiome-targeted medicine. Although obesity represents a particularly attractive application due to the growing recognition of microbiota-mediated metabolic dysfunction [202], the principles underlying phage-based microbiota modulation may be broadly applicable to numerous metabolic and inflammatory disorders in which specific bacterial populations contribute to disease development [228]. As our understanding of microbial ecology and host–microbiome interactions continue to expand, bacteriophages have the potential to become a cornerstone technology for the next generation of targeted nutritional and therapeutic interventions.

## 11. Conclusions

Available evidence supports the involvement of the gut microbiota in obesity through alterations in its composition, functional capacity, and communication with the host, which may contribute to metabolic and inflammatory processes associated with the disease. Although conventional strategies, including dietary interventions, prebiotics, probiotics, and fecal microbiota transplantation, have demonstrated the ability to modulate the gut ecosystem, their efficacy remains variable, and limitations persist in achieving consistent changes in the microbiota and metabolic outcomes. In this context, identifying microbial populations and functions with demonstrated relevance to obesity-associated mechanisms may provide a foundation for more selective microbiome interventions. Among these, bacteriophages represent a promising tool due to their ability to selectively target bacterial populations while potentially limiting alterations to other components of the microbial community. However, their therapeutic application still faces challenges related to target identification, bacterial resistance, phage stability and persistence, as well as safety, efficacy, and clinical validation. Importantly, direct evidence supporting the efficacy of phage therapy for obesity remains insufficient, and its clinical application cannot yet be considered established. Future research should prioritize causal validation of strain-level targets, assessment of metabolic and inflammatory outcomes in relevant preclinical models, optimization of phage formulations and dosing, and rigorous evaluation of safety and efficacy in controlled human trials.

## Figures and Tables

**Table 1:** Variability in the association between the Bacillota/Bacteroidota ratio and obesity across human studies.

Population/Comparison Groups	Microbiota Analysis	Main Finding	B/B–Obesity Association	Covariate Adjustment/Confounder Control	Ref.
Spanish adults with overweight/obesity (*n* = 96; BMI 25–35 kg/m^2^) vs. normal-weight controls (*n* = 32; BMI <25 kg/m^2^)	16S rRNA (V3-V4), Illumina MiSeq	Higher B/B ratio in overweight/obesity.	↑ Increased	B/B analysis: unadjusted. Confounder control: recent antibiotic use and weight-loss pharmacotherapy excluded; age differed between groups.	[46]
Sardinian adults with overweight/obesity (*n* = 46; BMI ≥25 kg/m^2^) vs. normal-weight controls (*n* = 46; BMI 18.5–24.9 kg/m^2^)	16S rRNA (V3-V4), Illumina MiSeq	Higher B/B ratio in overweight/obesity.	↑ Increased	B/B analysis: unadjusted. Confounder control: age, sex, and smoking considered in separate multivariate microbial analyses; recent antibiotic, PPI, probiotic, and prebiotic use excluded.	[47]
Chinese adults with obesity (*n* = 21; observed BMI 31.4–49.5 kg/m^2^) vs. controls (*n* = 21; observed BMI 16.5–25.4 kg/m^2^)	16S rRNA (V3-V4), Illumina MiSeq	Lower B/B ratio in obesity.	↓ Decreased	B/B analysis: no adjusted model reported. Confounder control: recent antibiotic use excluded for obesity group; controls from independent dataset; age and sex differed between groups.	[30]
Italian children/adolescents with obesity (*n* = 55; BMI-SDS >+2; severe obesity >+3) vs. normal-weight controls (*n* = 25; WHO age-/sex-specific criteria)	16S rRNA (multiple regions), Ion GeneStudio S5	Lower B/B ratio in obesity.	↓ Decreased	B/B analysis: no adjusted model identified. Confounder control: age-/sex-matched controls; recent antibiotic and probiotic use excluded.	[48]
Emirati adults with obesity (*n* = 43; BMI ≥35 kg/m^2^) vs. normal-weight controls (*n* = 31; BMI 18.5–24.9 kg/m^2^)	16S rRNA (V3-V4), Illumina MiSeq	No significant difference in B/B ratio between groups.	↔ No significant association	B/B analysis: no adjusted model reported. Confounder control: age-/sex-matched recruitment; recent antibiotic use excluded.	[49]
Uygur adults with obesity (*n* = 27; BMI ≥28 kg/m^2^) vs. normal-weight controls (*n* = 20; BMI 18.5–24 kg/m^2^)	16S rRNA (V3-V4), Illumina MiSeq	No significant difference in B/B ratio between groups.	↔ No significant association	B/B analysis: unadjusted group comparison. Confounder control: recent antibiotic use and selected metabolic medications excluded.	[50]
Mexican adults with obesity (*n* = 38; BMI ≥30 kg/m^2^) vs. controls (*n* = 27; BMI classification not specified)	16S rRNA (V3-V4), Illumina MiSeq	No significant difference in B/B ratio between groups.	↔ No significant association	B/B analysis: no adjusted model identified. Confounder control: age and sex compared between groups; additional controls not established for B/B analysis.	[51]
Mexican adults with obesity (*n* = 10; BMI 30–34.9 kg/m^2^), overweight (*n* = 10; BMI 25–29.9 kg/m^2^), and normal weight (*n* = 10; BMI 19.5–24.9 kg/m^2^)	16S rRNA (V3-V4), Illumina MiSeq	Higher B/B ratio was associated with higher BMI.	↑ Increased	B/B analysis: no adjusted model reported. Confounder control: recent antibiotic, antifungal, probiotic, and prebiotic use excluded.	[33]
Croatian adults across body-weight categories (*N* = 151; overweight: BMI ≥25 kg/m^2^; normal weight: BMI 18.5–24.9 kg/m^2^; underweight: BMI <18.5 kg/m^2^)	16S rRNA sequencing, Illumina MiSeq	No significant association between B/B ratio and excess body weight.	↔ No significant association	B/B analysis: unadjusted logistic regression. Confounder control: demographic, dietary, lifestyle, and medication variables considered in separate multivariable analyses.	[52]

BMI: body mass index; BMI-SDS: body mass index standard deviation score; PPI: proton pump inhibitor; WHO: World Health Organization.

**Table 2:** Experimental evidence linking specific gut bacterial taxa to obesity, metabolic dysfunction, and chronic low-grade inflammation and their proposed mechanisms of action.

Bacterial Taxon (Strain)	Experimental Model	Main Findings	Proposed Mechanisms	Ref.
*Akkermansia muciniphila*	High-fat diet (HFD)-induced obesity mouse model.	Lower abundance was associated with obesity progression, adipose tissue inflammation, insulin resistance, hyperglycemia, hyperinsulinemia, and altered lipid metabolism.	Negative correlation with inflammatory markers; positive association with adipose tissue browning markers and lipid oxidation pathways.	[53]
*Akkermansia muciniphila*	HFD mouse model with oral bacterial supplementation.	Administration reversed fat mass gain, metabolic endotoxemia, adipose tissue inflammation, and insulin resistance in obese mice.	Increased endocannabinoid production, improved gut barrier integrity, enhanced mucus layer thickness, and reduced circulating LPS.	[54]
*Akkermansia muciniphila* (live and pasteurized)	Randomized double-blind placebo-controlled clinical trial.	Improved insulin sensitivity, reduced insulinemia, plasma cholesterol, body weight, and markers of liver dysfunction and inflammation in overweight/obese subjects.	Reduced systemic inflammation and improved metabolic homeostasis without major alterations of global microbiome structure.	[55]
*Christensenella minuta*	Germ-free mice receiving microbiota transplantation amended with *C. minuta*.	Reduced weight gain and altered microbiome composition after transplantation into recipient mice.	Restructuring of the gut microbial community associated with a lean phenotype.	[56]
*Christensenella minuta* DSM33407	Diet-induced obesity mouse model and SHIME ex vivo simulator.	Protected against diet-induced obesity, reduced glycemia and leptin levels, and improved metabolic markers.	Inhibited hepatic de novo lipogenesis, maintained gut epithelial integrity, and decreased Bacillota/Bacteroidota ratio.	[57]
*Parabacteroides distasonis*	HFD-induced obesity mouse model.	Prevented obesity, improved insulin sensitivity, alleviated NAFLD, and reduced metabolic dysfunction.	Production of succinate and secondary bile acids; activation of intestinal gluconeogenesis and FXR signaling; improved gut barrier function.	[58]
*Blautia wexlerae*	Human cohort analysis and HFD mouse supplementation experiments.	Reduced obesity and type 2 diabetes-associated phenotypes and improved metabolic dysfunction.	Production of S-adenosylmethionine, acetylcholine, L-ornithine, succinate, acetate, and lactate; anti-inflammatory effects and remodeling of gut microbiota composition.	[59]
*Ruminococcus gnavus*	In vitro mechanistic studies and immune cell stimulation assays.	Promoted inflammatory responses and is enriched during inflammatory disease states.	Produces a glucorhamnan polysaccharide that stimulates dendritic cells to secrete TNF-α through TLR4 signaling.	[63]
*Enterobacter cloacae* B29	Germ-free mice mono-associated with strain B29 under HFD.	Induced obesity, insulin resistance, elevated endotoxemia, and systemic inflammation.	Production of endotoxin (LPS), resulting in increased circulating endotoxin load and inflammation.	[64]
*Bacteroides vulgatus* Bv46	HFD-induced hyperlipidemic rat model.	Reduced body weight gain, improved serum lipid profile, lowered inflammatory cytokines, and ameliorated hyperlipidemia.	Bile salt hydrolase activity, increased fecal bile acid excretion, enhanced SCFA production (propionate and butyrate), and modulation of gut microbial composition.	[60]
*Faecalibacterium prausnitzii* ATCC 27766	High-fat diet mouse model with oral administration.	Reduced adipose tissue inflammation, improved insulin sensitivity, decreased hepatic fat accumulation, and enhanced hepatic health.	Increased adiponectin signaling, enhanced fatty acid oxidation, improved intestinal barrier function, and production of anti-inflammatory metabolites.	[61]
*Roseburia intestinalis*	TNBS-induced colitis mouse model, TLR5-deficient mice, and in vitro epithelial–immune cell coculture assays.	Reduced intestinal inflammation and promoted anti-inflammatory immune responses.	Activation of TLR5 signaling in intestinal epithelial cells, induction of TSLP production, stimulation of IL-10 and TGF-β secretion by dendritic cells, and expansion of regulatory T cells (Tregs).	[62]

FXR: farnesoid X receptor; LPS: lipopolysaccharide; NAFLD: non-alcoholic fatty liver disease; SCFA: short-chain fatty acid; SHIME: Simulator of the Human Intestinal Microbial Ecosystem; TNBS: 2,4,6-trinitrobenzenesulfonic acid; TSLP: thymic stromal lymphopoietin.

## Data Availability

No new data were created or analyzed in this study. Data sharing is not applicable to this article.
